# Reactivation Process of Activated Carbons: Effect on the Mechanical and Adsorptive Properties

**DOI:** 10.3390/molecules25071681

**Published:** 2020-04-07

**Authors:** Rita B. Cevallos Toledo, Carlos F. Aragón-Tobar, Sebastián Gámez, Ernesto de la Torre

**Affiliations:** Department of Extractive Metallurgy, Escuela Politécnica Nacional, Quito 170517, Ecuador; rita.belen@hotmail.com (R.B.C.T.); carlos.aragont@epn.edu.ec (C.F.A.-T.); sebastian.gamez@epn.edu.ec (S.G.)

**Keywords:** reactivation, activated carbon, mechanical properties, adsorption

## Abstract

Carbon reactivation is a strategy to reduce waste and cost in many industrial processes, for example, effluent treatment, food industry, and hydrometallurgy. In this work, the effect of physical and chemical reactivation of granular activated carbon (AC) was studied. Spent activated carbon (SAC) was obtained from a carbon in pulp (CIP) leaching process for gold extraction. Chemical and physical reactivations were evaluated using several acid-wash procedures (HCl, HNO_3_, H_2_SO_4_) and thermal treatment (650–950 °C) methods, respectively. The effect of the reactivation processes on the mechanical properties was evaluated determining ball pan hardness and normal abrasion in pulp resistance. The effect on the adsorptive properties was evaluated via the iodine number, the gold adsorption value (*k* expressed in mg Au/g AC), and Brunauer–Emmett–Teller (BET) surface area. Initial characterization of the SAC showed an iodine number of 734 mg I_2_/g AC, a *k* value of 1.37 mg Au/g AC, and a BET surface area of 869 m^2^/g. The best reactivation results of the SAC were achieved via acid washing with HNO_3_ at 20% *v*/*v* and 50 °C over 30 min, and a subsequent thermal reactivation at 850 °C over 1 h. The final reactivated carbon had an iodine number of 1199 mg I_2_/g AC, a *k* value of 14.9 mg Au/g AC, and a BET surface area of 1079 m²/g. Acid wash prior to thermal treatment was critical to reactivate the SAC. The reactivation process had a minor impact (<1% change) on the mechanical properties of the AC.

## 1. Introduction

Activated carbon (AC) has excellent adsorbent properties owing to its large specific surface area (1000–1500 m^2^/g), which indicates a large number of active sites [1]. AC is extensively used in several industries like pharmaceutical, chemical, metallurgical, and petroleum, among others. Additionally, AC has important applications in water treatment, both prior to distribution and before effluent discharge from industries, as well as treatment of flue gases [1,2,3]. Specific industrial applications of AC are removal of dyes, adsorption, and concentration of heavy metals [1,4,5,6,7]. The removal of dyes from industrial waters avoids contamination of water bodies, complying with environmental regulations. In addition to the removal of contaminants such as heavy metals, AC in the industry allows the concentration of precious metals like gold and silver. Hence, AC is widely employed in the mining and metallurgical industry.

In Ecuador, gold mining has greatly increased during the last decade owing to government stimulus and global demand [8,9]. The gold reserves of the country are estimated at 8.5 million ounces [8], and there is a high demand for activated carbon because gold recovery techniques are migrating from traditional amalgamation to a more technical process, like cyanidation leaching and AC adsorption process [8]. The typical gold extraction process has multiple stages: ore comminution process (crushing and milling), leaching, AC adsorption, AC elution, electro-winning or zinc precipitation, and gold refining [10,11]. Leaching is achieved typically with a solution of NaCN; the ${\mathrm{CN}}^{-}$ ion complexes gold to form an aurocyanide complex, which remains dissolved in the solution. Subsequently, AC in the carbon in pulp process (CIP) absorbs and concentrates the aurocyanide complex. The gold is desorbed from the loaded AC by an elution process, and it is recovered as metal via electrolysis. Once the elution is over, AC goes to a reactivation process that regenerates its adsorptive capabilities, and this regenerated AC is reused in the CIP process [1,10,11].

The gold extraction industry is a very profitable business worldwide. It involves the use of large amounts of activated carbon, especially in medium and large capacity operations. AC is used owing to the high gold recovery reached during the CIP process. However, the gold adsorption capacity of AC decreases after several batch cycles owing to the blockage of the carbon pores caused by the progressive deposition of clays, oxides, and hydroxides of calcium and magnesium on the AC surface. Consequently, the reactivation process is not only essential to restore the adsorption capacities of the carbon, but also it could improve the economy of the process by reducing the amount of virgin carbon needed [10,12,13,14].

Some of the most used reactivation procedures include the following: acid-wash, treatment with supercritical fluids, microwave reactivation, electrochemical process, and thermal reactivation. Previous studies concluded that, in order to achieve a better reactivation degree, it is critical to combine acid-wash with thermal reactivation of the spent activated carbon (SAC). Thus, whereas acid-wash removes inorganic pollutants by dissolving them, thermal reactivation eliminates organic waste by thermal decomposition [15,16,17,18,19,20,21,22,23,24,25,26,27].

The variation in some properties of activated carbon after reactivation has been investigated in previous studies. Compared with SAC, the moisture and the ash content of AC subjected to a reactivation process decreased [28,29]. In order to be reused in the CIP process, reactivated carbon should have a value of ash content between 2% and 4% [29]. After reactivation, the iodine number and the Brunauer–Emmett–Teller (BET) specific surface area of AC increased significantly [13,15,28,29]. For instance, a carbon subjected to a chemical-thermal reactivation increased the iodine number from 534 mg I_2_/g CA before the treatment to 1070 mg I_2_/g CA after the reactivation [15]. If the activated carbon is subjected to a thermal reactivation, the iodine number increased with the rise in temperature [15,25,30]. Another important property of an activated carbon is the gold adsorption value *(k)*, which should be between 15 and 25 mg Au/g AC in order to use the carbon satisfactorily in the CIP process [15,26,30].

The cost of the virgin carbon (Calgon GCR-20) used in this study is $3.5/kg. According to our estimations, the cost of reactivation rises up to $0.5 per kg of SAC, based on the quantity of acid required for the acid wash and the energetic cost associated to maintain the temperature needed in the furnace during the thermal reactivation. Therefore, reactivated carbon is preferred over virgin carbon because reactivation constitutes a viable alternative to significantly reduce operative costs in the CIP process.

The innovative point of this research lies in the combination of two traditional reactivation processes (chemical and thermal reactivation). These reactivation methods are already known, but combining them brings interesting new insights into the advantages and limitations of each reactivation process. Additionally, there is already some empirical knowledge about the effectiveness of chemical reactivation using inorganic acids (HCl, HNO_3_, H_2_SO_4_). However, not all the acids would reach the same efficiency, limiting its application at a bigger scale. Thus, presenting the efficiency of these three acids would contribute to better understanding some of the empirical results concerning the chemical activation of activated carbon.

Activated carbon loses its efficiency by the progressive deposition of calcium salts on the pores during the CIP process. In industrial plants having the CIP process, SAC is firstly removed and sieved in order to eliminate fine carbon. Then, there is a next stage of reactivation, mainly chemical or thermal. At the industrial scale in Ecuador, sometimes, the choice of one of these treatments is done using mainly empirical criteria. In the case that chemical reactivation is chosen, the cheaper acid available is selected, but it could not always be the more efficient one. Therefore, in this study, the influence of each inorganic acid (HCl, HNO_3_, H_2_SO_4_) is tested, as an original approach to validate the empirical findings currently extended in the chemical reactivation that follows the CIP process at the industrial scale. Another aspect to highlight in this study corresponds to the hardness of AC. In fact, during the CIP process, there is a fine fraction of carbon lost in the tails owing to the abrasion caused by the agitation. So, as a part of the reactivation process proposed in our study, it is also necessary to quantify the hardness of SAC. Like this, only a minimum decrease in the hardness value of SAC is tolerated to occur in order to make the reactivation process technically viable.

The aim of this work is to assess the effects of chemical and thermal reactivation on the AC adsorptive and mechanical properties in order to suggest a reactivation procedure that allows the reuse of SAC in the CIP process.

## 2. Results and Discussion

### 2.1. Characterisation of Virgin Carbon and SAC

As a first stage, virgin carbon and SAC were characterized through properties, such as d_80_, iodine number, specific surface area, and ball-pan hardness, which are presented in Table 1.

Table 1 compares the characteristics of virgin carbon (*d_80_* = 3.1 mm) and SAC (*d_80_* = 2.5 mm). The main change observed corresponded to the iodine number and to the specific surface. These two parameters reported in SAC (734 mg I_2_/g AC and 869 m^2^/g) decreased by 15%–30% compared with the same parameters in virgin carbon (1036 mg I_2_/g AC and 1012 m^2^/g). This decrease in iodine number and specific surface area in SAC responded to the saturation by inorganic and organic pollutants, which block the porous decreasing the adsorptive capabilities of the AC compared with a virgin carbon. The ball-pan hardness remained at the same value of 98% for both carbons. Elemental analysis of virgin carbon and SAC (Appendix A, Table A1) revealed a similar content of the main components in both carbons (82% of C, 3% of H, 0.3% of N, and 6% of O).

### 2.2. Acid Wash (Chemical Reactivation)

**Figure 1:**
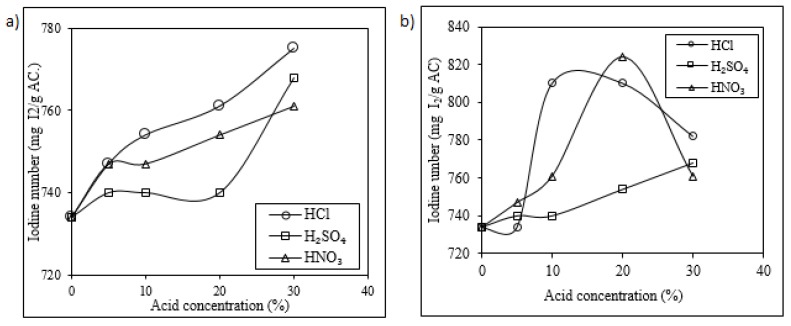
Influence of the concentration (5%, 10%, 20%, and 30% *v*/*v*) of acids (HCl, H_2_SO_4_, and HNO_3_) on the iodine number (mg I_2_/g AC) at (**a**) 18 °C and (**b**) 50 °C for spent activated carbon (SAC) subjected to a chemical reactivation by acid wash.

The first reactivation treatment tested in this study was the chemical reactivation. This reactivation encompassed the acid wash at two temperatures (18 °C and 50 °C) using three inorganic acids (HCl, H_2_SO_4_, and HNO_3_) at four different concentrations (5%, 10%, 20%, 30% *v*/*v*). Figure 1 presents the influence of the concentration of these acids on the iodine number of the SAC after acid-wash at 18 °C and 50 °C, respectively.

As presented in Figure 1, the value of the iodine number increased with the increase of the acid concentration (from 5% *v*/*v* to 30% *v*/*v*) and with the increase of the temperature (from 18 to 50 °C); ranging from 735 to 775 mg I_2_/g AC at 18 °C and from 734 to 824 mg I_2_/g AC at 50 °C.

When the acid wash was performed with HCl (10% *v*/*v*) and a temperature of 50 °C, the iodine number reached a maximum of 810 mg I_2_/g AC, however, for more concentrated solutions (20% and 30% *v*/*v*), the value of the iodine number decreased (e.g., 782 mg I_2_/g AC using 30% *v*/*v* HCl). This behavior could be explained regarding the boiling point of the concentrated HCl solutions (~48 °C). Thus, we hypothesized that, at 50 °C, the solution begins to boil in the surface of activated carbon, generating gas pressure, which breaks the pores of the carbon, decreasing the iodine number. This concept about the destruction of the porous structure by gas generated inside the carbon porous has also been briefly mentioned in previous studies [31,32].

Similarly, at 50 °C, a maximum value of iodine number (824 mg I_2_/g AC) was reached at a concentration of 20% *v*/*v* of HNO_3_, and then it decreased to 761 mg I_2_/g AC at a concentration of 30% *v*/*v* of HNO_3_. This tendency may be explained by the reaction of HNO_3_ with the carbon, generating CO_2_ (Equation (1)). This reaction causes a breakdown of the porous structure, thereby reducing the iodine number.

4HNO_3_+C→ CO_2_+4NO_2_+2H_2_O. (1)

**Figure A1:**
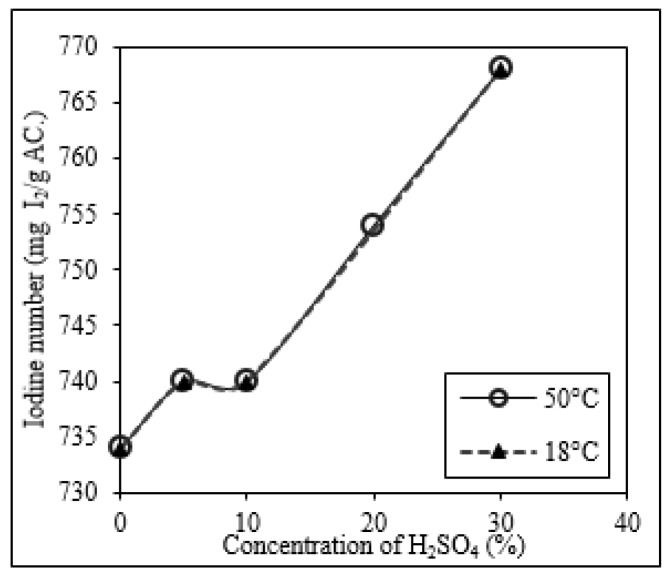
Influence of the concentration of H_2_SO_4_ (5%, 10%, 20%, and 30% *v*/*v*) on the iodine number (mg I_2_/g AC) of SAC subjected to chemical reactivation at 18 °C and 50 °C.

For the H_2_SO_4_ acid wash at 18 °C, similar values of iodine number were obtained compared with acid-wash at 50 °C, as is also presented in Figure 1 (a comparison between the iodine numbers obtained at these two temperatures for acid wash with H_2_SO_4_ is presented in Appendix B, Figure A1). Regarding the high boiling temperature of H_2_SO_4_ (337 °C) compared with the tested temperatures used during the acid wash, there is a low possibility of collapse of the carbon structure owing to the boiling of the acid solution inside it. Then, this lack of dependence within the range of temperatures examined may be explained by the solubility of the salts generated from H_2_SO_4_. In fact, a higher solubilization of the precipitated salts on the SAC surface would increase the iodine number. However, there is an insignificant variation in the solubility of the salts generated from H_2_SO_4_ at the two temperatures. For instance, the CaSO_4_ has a solubility of 0.2090 g/100 g H_2_O at 18 °C and 0.2097 g/100 g H_2_O at 50 °C [33].

**Figure 2:**
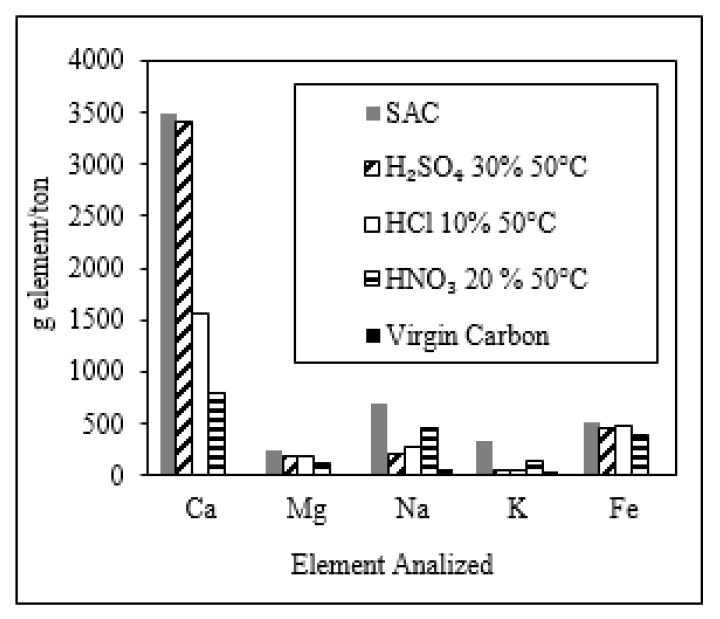
Content of Ca, Mg, Na, K, and Fe (g/ton) for reactivated carbons subjected to chemical reactivation at 50 °C with HCl (10% *v*/*v*), HNO_3_ (20% *v*/*v*), and H_2_SO_4_ (30% *v*/*v*), respectively. The content of these elements (Ca, Mg, Na, K, and Fe) is shown as well for virgin carbon and SAC.

Summarizing, SAC treated with acid wash at 50 °C reached higher values of iodine number when the treatment was performed with the following concentrations of the tested acids: HNO_3_ (20% *v*/*v*), HCl (10% *v*/*v*), and H_2_SO_4_ (30% *v*/*v*), respectively. Additionally, in Figure 2, the influence of the acid wash on the content of Ca, Mg, Na, K, and Fe in the carbon is presented. After the acid wash treatment, there was a decrease in the content of these elements compared with SAC. From these elements, Ca deserves special attention as it is added into the cyanide process in which Ca(OH)_2_ (slaked lime) is used as pH regulator. Thus, a better solubilization of Ca during the acid wash increases the iodine number. The highest value of Ca removal (up to 77%) was reached when HNO_3_ (20% *v*/*v*) was used. The acid wash using HNO_3_ (20% *v*/*v*) was more efficient as the solubility of the calcium nitrate is higher compared with the solubility of the calcium chloride and calcium sulfate [33]. Previous studies have performed acid washing of SAC only with HCl [15].

### 2.3. Thermal Reactivation

**Figure 3:**
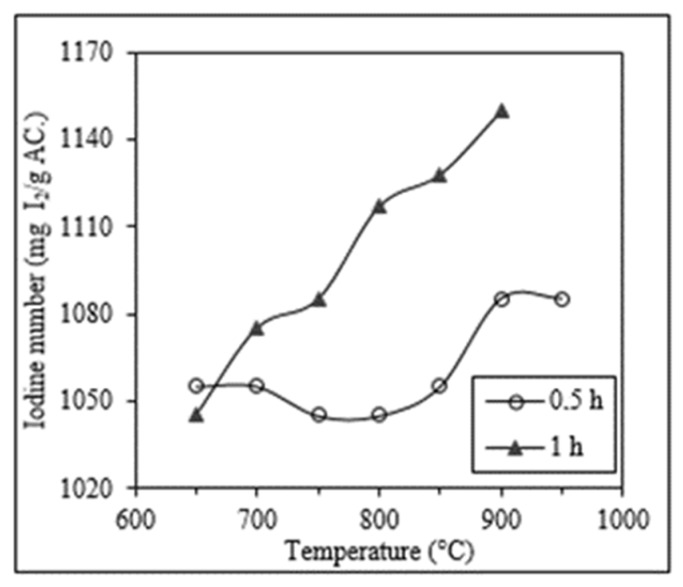
Influence of the reactivation time (0.5 and 1 h) on the iodine number (mg I_2_/g AC) of a SAC washed with H_2_SO_4_ (30% *v*/*v* at 50 °C) subjected to a thermal reactivation at different temperatures (from 650 °C to 950 °C).

The efficiency of the thermal reactivation process was analyzed based on two parameters: the reactivation time and the reactivation temperature (T_RA_). To illustrate the influence of the reactivation time on the iodine number, Figure 3 presents the variation of the iodine number of a thermally reactivated carbon previously washed with H_2_SO_4_ (30% *v*/*v* and 50 °C) in the function of the reactivation time (0.5 and 1 h). In this case, the iodine number increased from 1055 to 1085 mg I_2_/g AC when the thermal reactivation (*T_RA_* = 700 °C) lasted 0.5 h and 1 h, respectively. In the same way, with a reactivation temperature of 900 °C, an increase in the reactivation time from 0.5 to 1 h rendered an increase in the iodine number from 1085 to 1128 mg I_2_/g AC, respectively. The same enhancement effect of the reactivation time on the iodine number was also observed in carbons subjected to thermal reactivation that were previously washed at 50 °C with the other two acids tested (HCl 10% *v*/*v* and HNO_3_ 20% *v*/*v*).

**Figure 4:**
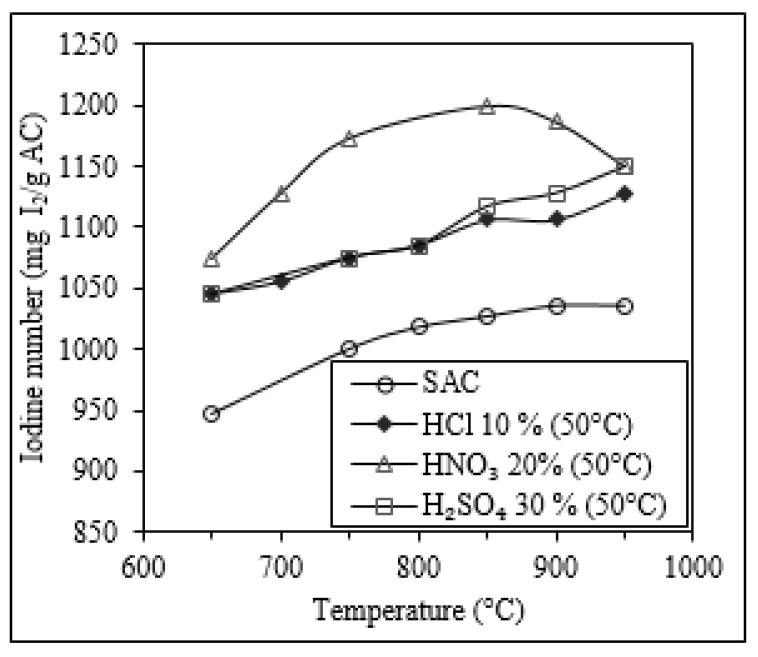
Influence of the reactivation temperature on the iodine number of SAC thermally reactivated during 1 h. Some of these carbons were subjected to a previous acid wash treatment (HCl 10% *v*/*v*, HNO_3_ 20% *v*/*v*, and H_2_SO_4_). SAC was only subjected to thermal reactivation.

Figure 4 shows the enhancement effect of the reactivation temperature (T_RA_) on the iodine number of carbons subjected to 1 h of thermal reactivation with and without a previous acid wash treatment. In general, the iodine number increased at a higher reactivation temperature, passing from 946 mg I_2_/g AC (*T_RA_* = 650 °C) to 1036 mg I_2_/g AC (*T_RA_* = 950 °C) in SAC without acid wash, from 1045 mg I_2_/g AC (*T_RA_* = 650 °C) to 1128 mg I_2_/g AC (*T_RA_* = 950 °C) in SAC washed with HCl (10% *v*/*v* −50 °C), from 1045 mg I_2_/g AC (*T_RA_* = 650 °C) to 1150 mg I_2_/g AC (*T_RA_* = 950 °C) in SAC washed with H_2_SO_4_ (30% *v*/*v* −50 °C), and from 1075 mg I_2_/g AC (*T_RA_* = 650 °C) to 1199 mg I_2_/g AC (*T_RA_* = 850 °C) in SAC washed with HNO_3_ (20% *v*/*v* −50 °C). However, the iodine number of SAC washed with HNO_3_ (20% *v*/*v* at 50 °C) decreased from 1199 mg I_2_/g AC (*T_RA_* = 850 °C) to 1150 mg I_2_/g AC (*T_RA_* = 950 °C). This decrease in the iodine number observed in SAC washed with HNO_3_ is explained by the oxidizing characteristic of this acid. During the acid wash, once HNO_3_ finishes reacting with the pollutants, it starts to react with the carbon, generating CO_2_, which breaks and weakens the porous structure (see Equation (1), Section 2.2). The variation of iodine number with temperature of carbon without reactivation was lower than that of the carbons acid-washed previously at all the temperatures tested, which confirms that the acid washing is necessary as a reactivation process [34], because it removes the inorganic compounds that block the pores.

Summarizing, carbon washed with HNO_3_ 20% *v*/*v* at 50 °C reactivated thermally at the temperature of 850 °C during 1 h reached the highest iodine number, with a value of 1199 mg I_2_/g AC. Our results confirmed that the iodine number increases with temperature and with the time of reactivation. These findings were in good accordance with previous studies [25,30]. The enhancement effect observed may be explained by the better mobilization of contaminants (e.g., humic acid, tar) from the carbon matrix owing to a higher dissolution and degradation of organic pollutants driven by the higher reactivation temperature and longer reactivation time.

### 2.4. Specific Surface Area

In order to better evaluate the performance of the carbon obtained by the reactivation process, not only is the iodine number required, but also the value of the specific surface area. Hence, the values of the specific surface area of virgin carbon, SAC without reactivation, and reactivated carbon are presented in Table 2.

After the CIP process, the specific surface area of carbon (SAC) (869 m^2^/g) decreased by 14% compared with the specific surface area of virgin carbon (1012 m^2^/g). When this carbon (SAC) was subjected to a reactivation process (acid wash at 50 °C with HNO_3_ 20% *v*/*v* and thermal reactivation during 1 h at 850 °C), the specific surface area of the reactivated carbon (1079 m^2^/g) increased and reached a similar value compared with the specific surface area of virgin carbon. Therefore, the enhancement effect of the reactivation process on the properties of the carbon is demonstrated. The reactivation process helped to removed contaminants from SAC, even making possible the creation of new pores on the carbon surface. The additional pores contributed to the increase in the value of specific surface, as has already been pointed out in previous studies [15].

In fact, as presented in Table 2, the difference between the values of specific surface area (SSA) of the virgin carbon and the reactivated carbon is only 67 m^2^/g (*SSA_reactivated_* = 1079 m^2^/g and *SSA_virgin_* = 1012 m^2^/g), which, according to our experience, is not as high as expected in order to notice a true difference in the adsorption properties. Besides, both carbons were produced following the same activation procedure, so we stand that any change observed in the reactivated carbon compared with the virgin carbon is the result of the reactivation process.

Additionally, the mechanism of gold adsorption observed in carbon is not only dependent on the specific surface area, but also on other parameters, such as the presence of active sites on the surface of carbon, as well as the shape and volume of pores. Thus, based only on values of the specific surface area, we cannot predict if a carbon would have a higher gold recovery. In our study, we complemented the analysis of the adsorptive properties of the carbon through the iodine number, the methylene blue index, the sugar discoloration index, pore distribution, and scanning electron microscopy (SEM) images (see Section 2.9).

### 2.5. Reactivation Sequence

In order to determine the best reactivation sequence of chemical and thermal reactivation, two procedures were performed: chemical reactivation followed by a thermal reactivation, and vice versa (thermal-chemical). These reactivation tests were performed considering the conditions in which the iodine number reached the highest values (acid wash at 50 °C with HCl 10% *v*/*v*, HNO_3_ 20% *v*/*v*, H_2_SO_4_ 30% *v*/*v*, and thermal reactivation during 1 h at 850 °C). The values of the iodine number of several carbons subjected to different sequences of reactivation are presented in Table 3.

The highest values of iodine number were reached when chemical reactivation took place prior to thermal reactivation. The sequence of chemical-thermal treatment produced carbons with a higher iodine number (1085–1199 mg I_2_/g AC) compared with reactivated carbons produced by the inverse sequence of thermal-chemical treatment (1035–1055 mg I_2_/g AC)_._ The main advantage of starting with the chemical treatment lies in a better dissolution and removal of the inorganic pollutants. On the contrary, if thermal reactivation is conducted first, inorganic pollutants could form insoluble salts by sintering. The sintering of the inorganic pollutants on the AC surface limits the efficiency of the reactivation procedure by passivation of the AC surface, as mentioned in previous studies [34].

### 2.6. Ball-Pan Hardness and Normal Abrasion in Pulp Resistance

The influence of the reactivation procedure on the mechanical properties of the carbons was evaluated through two parameters: ball-pan hardness and normal abrasion in pulp resistance. The percentages of ball-pan hardness and normal abrasion in pulp resistance were compared between virgin carbon, SAC, and three reactivated carbons. These results are presented in Table 4.

As can be observed in Table 4, the reactivation processes did not affect the mechanical properties of activated carbon. The value of ball-pan hardness among the tested carbons was 98%, showing no significant variation between virgin carbon, SAC, and the reactivated carbons. The value of normal abrasion in pulp resistance was 97% for SAC and for the chemically and chemical-thermal reactivated carbons. However, in the case of virgin carbon, this value was lower (93%). This lower value reflected the morphology of the virgin carbon in which sharp edges more prone to abrasion are still found compared with the SAC carbons. The thermally reactivated carbon showed the lowest value of abrasion (90%), exhibiting a debilitating effect on the carbon structure induced by heat. The performance of the carbons in the abrasion in the pulp resistance test was lower compared with that in the ball-pan hardness test. This difference is explained considering that, in the abrasion in the pulp resistance test, the auriferous mineral used (see Appendix C, Table A2) was highly abrasive owing to its content of quartz (86%).

### 2.7. Gold Adsorption Isotherms

**Figure 5:**
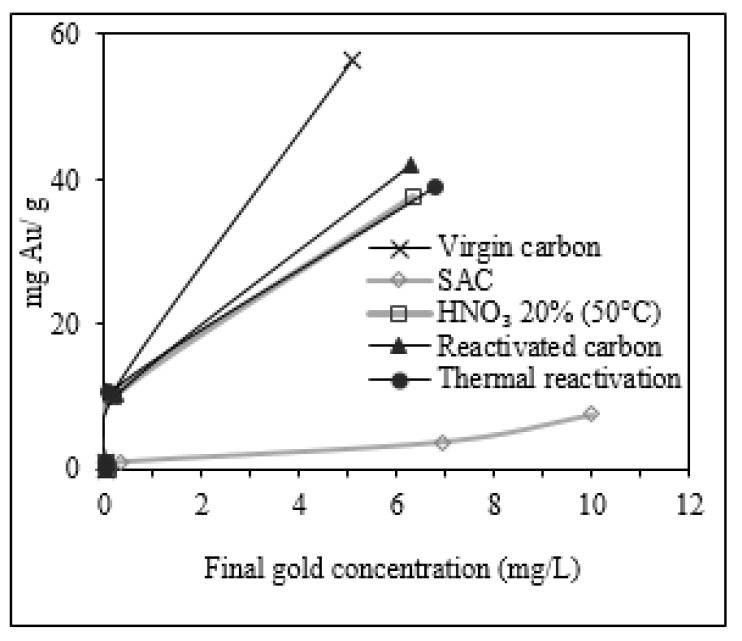
Gold adsorption isotherms of virgin carbon, SAC, and reactivated carbons (chemically, thermally, and chemical-thermally).

The performance of the reactivated carbon produced was evaluated by the gold adsorption capacity of this carbon compared with that of a virgin carbon. In Figure 5, adsorption isotherms for several carbons are shown. These adsorption isotherms fitted the Freundlich model. The value of the adsorption equilibrium constant k was determined at a final gold concentration of 1 mg/L [10]. Virgin carbon presented the best gold adsorption with a *k* value of equilibrium of 18.45 mg Au/g AC. The lowest adsorption was attained for the SAC (carbon without reactivation), which had a *k* value of 1.37 mg Au/g AC. This low value exhibits a limited gold adsorption owing to the saturation of the pores in this used carbon. Chemically reactivated carbon had a *k* value of equilibrium of 13.63 mg Au/g AC, whereas the *k* value of equilibrium of thermally reactivated carbon was 13.18 mg Au/g AC. Compared with these reactivated carbons, the chemical-thermally reactivated carbon exhibited a higher *k* value of equilibrium of 14.9 mg Au/g AC. Therefore, the combined reactivation process (chemical-thermal) restored the adsorption capabilities in the SAC, making it suitable again for the CIP process (acceptable range in the CIP process: 15–25 mg Au/g AC [35]). Nevertheless, the reactivated carbon did not reach the same *k* value of equilibrium of virgin carbon. In fact, the reactivated carbon had a residual gold content of 27.35 mg Au/g, which disrupted the balance of adsorption, rendering it impossible for the reactivated carbon to attain the same adsorption capability of a virgin carbon.

This residual gold on the carbon appears as a result of the adsorption/desorption equilibrium during the elution process. Thus, the presence of this residual gold on reactivated carbon would limit the quantity of gold adsorbed compared with virgin carbon, which contains no residual gold. Therefore, considering the adsorption/desorption equilibrium, the gold recovery in reactivated carbon will be lower compared with that in virgin carbon, even though both carbons showed similar values of specific surface area (see Table 2). In order to recover all the gold adsorbed in the carbon, a total combustion of the carbon would be envisaged, which is impractical.

### 2.8. Gold Adsorption Kinetics in CIP Process

**Figure 6:**
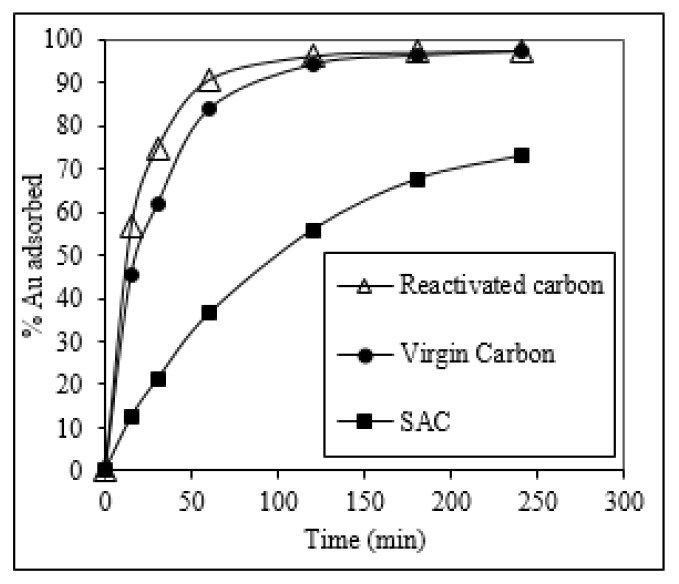
Gold adsorption kinetics (%Au adsorbed as function of time) of virgin carbon, SAC, and reactivated carbon (acid-wash HNO_3_ 20% *v*/*v* at 50 °C and thermal reactivation at 850 °C and 1 h). These tests were conducted on the pulp obtained from the cyanidation of the auriferous ore (Pacto).

The performance of the carbon in a CIP process was evaluated by following the gold adsorption kinetics in a pulp obtained from the cyanidation of an auriferous ore from Ecuador (see Appendix C, Table A2). Virgin and reactivated carbons had similar reaction rates on the CIP process, as shown in Figure 6, with an equilibrium constant of 2.2 × 10^−2^ and 2.3 × 10^−2^ min^−1^, respectively. Both carbons attained a gold adsorption close to 100% after 4 h, which demonstrated that the proposed reactivation process was able to restore the adsorption capabilities of SAC. The improvement of the adsorption is a consequence of the reactivation process, which eliminates the pollutants that block the active sites, allowing them to be used in the CIP.

### 2.9. Comparison of the Properties of Virgin Carbon, SAC, and Reactivated Carbon

In order to better interpret the gold adsorption performance achieved by the reactivated carbon (see Section 2.8), a comparison of the main properties of the reactivated carbon with those of the virgin carbon becomes pertinent. Table 5 compares virgin carbon, SAC, and reactivated carbon based on physical properties, such as moisture content, volatile content, ash content, and fixed carbon. Additional information of the structure of the carbons was obtained comparing the iodine number, methylene blue index, and sugar discoloration index.

The moisture content of reactivated carbon (0.54%) is lower than the moisture content of the SAC (0.81%). The decrease in moisture content is a consequence of the thermal reactivation process [13]. In the reactivated carbon, the volatile content, as well the ash content, decreased after the reactivation process, reaching values of 6.10% and 3.35%, respectively. The ash content in the reactivated carbon is in good accordance with previous reactivation studies [28,29], which means that, based on this parameter, the reactivated carbon is suitable for the CIP process. The content of fixed carbon increased after the reactivation process from 80% to 89%, owing to the decrease in the ash content.

The iodine number of reactivated carbon (1199 mg I_2_/g AC) was higher than that reported in virgin carbon (1036 mg I_2_/g AC). The reactivated carbon was subjected to a thermal reactivation process, which not only removed volatile pollutants, but also created new porous. The methylene blue indexes were in the same range (27.9 mg/ 100g AC) for the three carbons reported, implying that the number of mesoporous remained the same. Similarly, sugar discoloration indexes were in the same range (~34 UBR (basic units of reference)) for the three reported carbons, suggesting that the macro porous structure remained unchanged during the reactivation process.

The reactivated carbon produced in this study had good macro, meso, and micro porosity, as the values of the iodine number, methylene blue index, and sugar discoloration index were similar to those reported for the virgin carbon (see Table 5). In fact, macro and meso porosity are necessary to allow the diffusion of the aurocyanide complex into the structure of the activated carbon, while micro porosity is the one that adsorbs the gold complex in order to be recovered.

**Figure A2:**
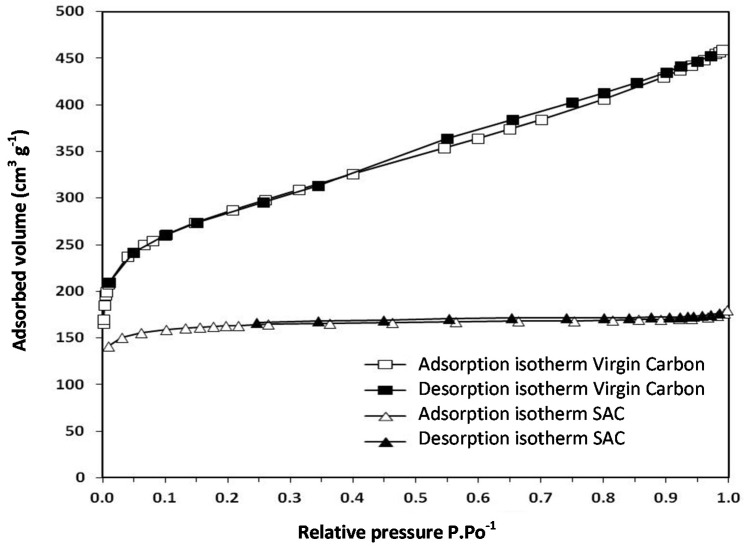
N_2_ adsorption–desorption isotherms of virgin carbon and SAC.

**Figure A3:**
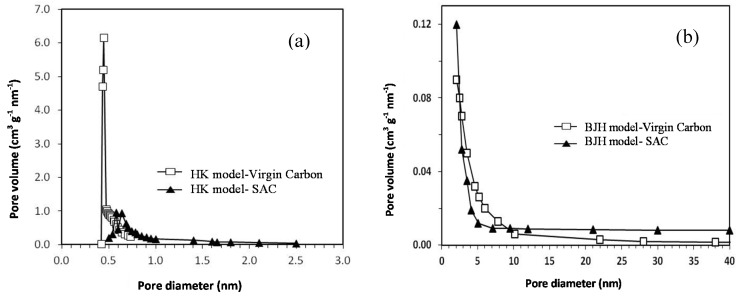
Pore diameter of virgin carbon and SAC calculated using the (**a**) Horvath, Kawazoe (HK) model and (**b**) Barrett, Joyner, Halenda (BJH) model.

Additionally, N_2_ adsorption isotherms of virgin carbon and SAC are included in Appendix D, Figure A2. As presented in Figure A2, the N_2_ adsorption–desorption isotherms of virgin carbon and SAC presented hysteresis type H4, which is normally observed on microporous adsorbents. The diameter of pores and micro pores was calculated following the HK (Horvath, Kawazoe) and BJH (Barrett, Joyner, Halenda) methods (Appendix D, Figure A3). Then, the average values obtained for virgin carbon and SAC were 0.7 and 0.6 nm for the micropores and 4.4 and 3.9 nm for the pores, respectively. It is important to notice that these results were relatively similar for all the samples analyzed, showing that the contribution of micro pores to the total surface area of the carbons tested in our study was 80%.

**Figure A4:**
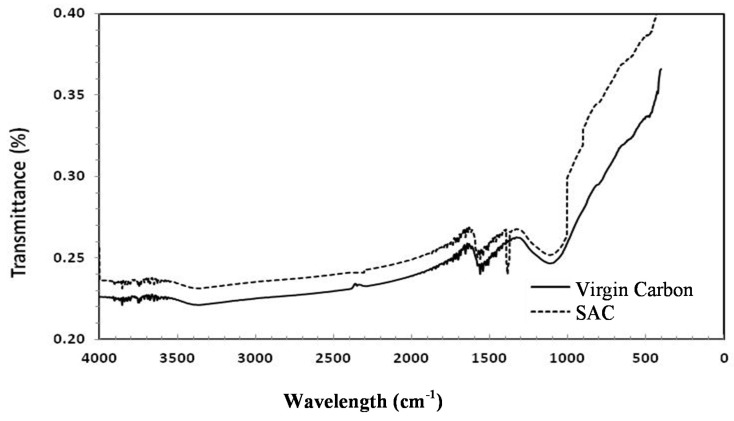
FTIR spectrum of virgin carbon and SAC.

FTIR results are presented in Appendix E, Figure A4. The functional groups on the surface of virgin carbon and SAC were identified by FTIR analysis (Appendix E, Table A3). The functional groups identified were similar to those mentioned by Bansal R. et al. [36] for Filtrasorb 200 activated carbon produced by physical activation of bituminous mineral carbon. Thus, these results indicated that the surface of the active carbon virgin and SAC has a strongly basic character, contains phenolic groups, and has a very low ion exchange capacity. Additionally, it is worth noting the presence of carbonate groups on the surface of SAC, which is proof of the blockage on the surface after the CIP process, contrasting with the virgin carbon, where this group is absent.

**Figure 7:**
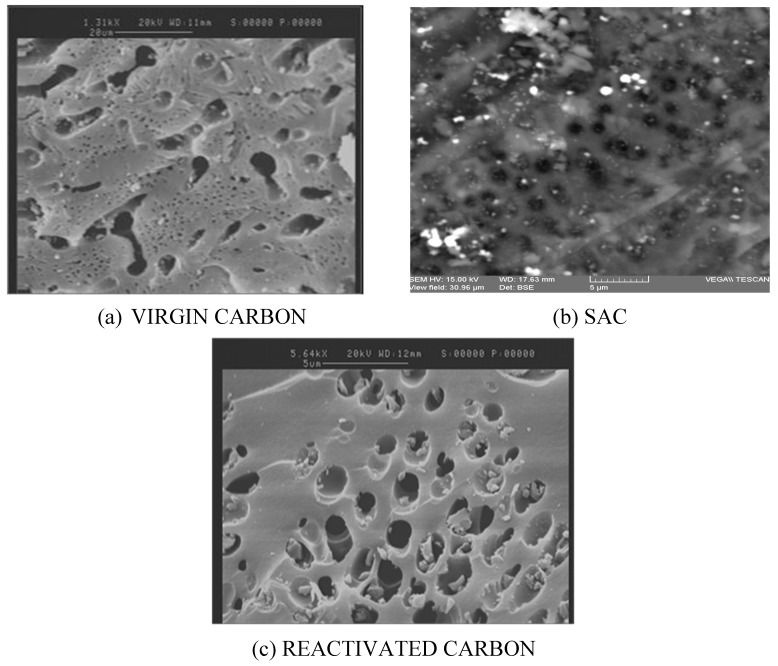
Scanning electron microscopy (SEM) images of (**a**) virgin carbon, (**b**) SAC, and (**c**) reactivated carbon. This image has been modified in order to highlight the deposits of calcium (on lighter color) on the surface. Reactivation conditions: acid-wash HNO_3_ 20% *v*/*v* at 50 °C and thermal reactivation at 850 °C and 1 h.

Certain structural characteristics of virgin carbon, SAC, and reactivated carbon were also analyzed by SEM. In Figure 7, it is shown that the porous structure remained more or less unaltered before and after the reactivation process. The presence of pores of around 1 µm and macro pores of different sizes is seen in the three images presented in Figure 7. In Figure 7a, an image of the virgin carbon is presented. In Figure 7b, the image of SAC is modified in order to better illustrate the salt deposits (on lighter color) on the carbon surface. Finally, Figure 7c displays the surface of carbon after the reactivation treatment, showing a good removal of the salt deposits that were previously blocking the surface of the SAC.

Summarizing, the properties of SAC after the reactivation process were similar to those of virgin carbon, making the reactivated carbon a suitable candidate for the CIP process. With the reactivation process, it was possible to eliminate the deposits on the carbon surface without entailing a significant loss neither in the gold absorptive properties nor in the mechanical properties.

## 3. Materials and Methods

### 3.1. Acid Wash (Chemical Reactivation) and Thermal Reactivation

For the acid wash, 100 g of the sample of carbon was stirred during 0.5 h with 100 mL of acid solution, and then the carbon was washed with Na(OH) (1% *v*/*v*) and rinsed with distillated water until the pH of washing water reached a value of 7. The procedure was performed at 18 °C (room temperature) and 50 °C, with HCl, H_2_SO_4_, and HNO_3_, modifying the concentration of the solution between 5% and 30% *v*/*v*. The iodine number was determined after each wash. The first temperature of 18 °C corresponded to the room temperature. For the choice of the second temperature, two phenomena were taken into account. The second temperature should be high enough to facilitate the solubilization of salts on the carbon surface, but not too high so as to provoke a considerable evaporation of the acid. Thus, a good compromise between these two conditions was fulfilled with a temperature of 50 °C for the chemical reactivation.

Regarding thermal reactivation, 15 g of sample of carbon in a closed crucible was heated in an electrical muffle at temperatures between 650 °C and 950 °C, during 0.5 and 1 h. The iodine number was determined after each reactivation.

### 3.2. Characterization of Carbon Without Reactivation (CIP), Virgin Carbon, and Reactivated Carbon

Each parameter was determined by the corresponding norm:

Moisture content (ASTM-D3173), volatile content (ASTM-D3175), fixed carbon and ashes (ASTM-D3174), sugar discoloration (NMX-F-299-1980), iodine number (AWWA B604-74/4.7), methylene blue index (ASTM C837), ball-pan hardness (ASTM 3802), gold adsorption capacity (AWWA B604-74/2.6), and specific surface area Brunauer–Emmett–Teller (BET).

The BET surface area was measured with a Quantachrome Instruments Nova4200e (Quantachrome Instruments, Boynton Beach, FL, USA). The specific surface area of carbon is best approached by inert gas (N_2_) adsorption using the Brunauer, Edward, and Teller method (or BET method) [37], as described in the standard ISO 9277:2010. The total surface area BET of activated carbons was calculated by a multi-point analysis of the BET isotherms. As microporosity is characteristic of activated carbons, pore volume and pore size distribution were determined using the HR (Horvath, Kawazoe) and BJH (Barrett, Joyner, Halenda) models [38,39].

For the analysis of carbon texture, a scattering electron microscopy analysis was performed with a Vega TESCAN microscope (TESCAN, Brno, Czech Republic). Qualitative characterization of activated carbons was performed by infrared analysis with a Perkin Elmer equipment Spectrum One (Perkin Elmer, Shelton, CT, USA). All tests were performed using KBr pills prepared with 0.15 mg of AC-O or CM mixed with 300 mg of KBr and dried overnight at 110 °C.

The content in ashes: Ca, Mg, Na, K, Fe, Cu, and Zn was determined by atomic absorption spectrometry, after calcination at 950 °C and acid digestion. These measurements were done with a Perkin Elmer A-Analyst 300 spectrometer (Perkin Elmer, Shelton, CT, USA).

### 3.3. Normal Abrasion in Pulp Resistance

This test was performed in order to determine the mechanical strength of activated carbon (−2.4 + 1.4 mm) in normal operation conditions. First, a pulp of 24% solids was prepared using an Ecuadorian auriferous ore (see Appendix C, Table A2). This pulp (2 L) was stirred with activated carbon (10 g per litter of pulp) during 24 h. Then, the carbon was recovered by sieving (+1.4 mm), and dried during 12 h at 110 °C. The final weight of the dried carbon was registered. Finally, by weight difference, the percentage of carbon lost was determined.

### 3.4. Gold Adsorption Isotherms

For gold adsorption isotherms, 1 L of cyanide solution was prepared with a content of NaCN of 1 g/L. Then, 10 mL of standard solution of gold with a concentration of 1000 mg/L was added to the solution, in order to attain a final gold concentration of 10 mg/L. Next, 0.01 g of carbon was added to 100 mL of the gold-cyanide solution and stirred during 24 h. This procedure was repeated, but incorporating 0.1, 1.0, and 10 g of carbon each time. After the adsorption time was reached, samples were taken in order to determine the gold content remaining in the solution by atomic absorption spectrometry with a Perkin Elmer A-Analyst 300 spectrometer (Perkin Elmer, Shelton, CT, USA).

### 3.5. Gold Adsorption Kinetics

The same gold-cyanide solution prepared for the isotherms test (Section 3.4) was used in the adsorption kinetics test. A sample of 0.1 g of carbon was stirred with 100 mL of gold-cyanide solution during 0.25, 0.5, 1, 2, and 3 h. After each time, samples of the solution were taken, in order to determine the gold content by atomic absorption spectrometry with a Perkin Elmer A-Analyst 300 spectrometer (Perkin Elmer, Shelton, CT, USA).

### 3.6. CIP Process

Firstly, the cyanidation of the auriferous mineral was conducted. The NaCN concentration and the content of gold in solution were verified at the following times: 0.5, 1, 2, 4, 6, 8, and 24 h. Once the cyanidation time concluded, 10 g of activated carbon per litter of pulp was added and the adsorption process started. The adsorption process finished after 4 h. The content of gold in solution was verified at 0.25, 0.5, 1, 2, 3, and 4 h.

## 4. Conclusions

The best experimental conditions for the reactivation of spent activated carbon (SAC) were achieved via acid washing with HNO_3_ at 20% *v*/*v* and 50 °C during 30 min and a subsequent thermal reactivation at 850 °C during 1 h. This reactivation process did not have much influence on the mechanical properties of carbon, as the ball-pan hardness of reactivated carbon compared with virgin carbon remained at 98%. Besides, the reactivated carbon produced in this study had good macro, meso, and micro porosity. In fact, the values of the iodine number (1199 mg I_2_/g AC), methylene blue index (27.9 mg/100g AC), and sugar discoloration index (34 basic units of reference) of the activated carbon were similar to those reported for the virgin carbon. The final reactivated carbon had a *k* value of 14.9 mg Au/g AC and a BET surface area of 1079 m²/g. Therefore, reactivated carbon obtained with the reactivation procedure proposed in this study can be used successfully in the carbon in pulp process (CIP).

The type of acid employed for the chemical reactivation plays an important role in the reactivation process. Chemical reactivation was performed using three acids (HCl, HNO_3_, and H_2_SO_4_). From these acids, the more appropriate acid for this process turned out to be HNO_3_ (20% *v*/*v* at 50 °C), because it reached the highest removal of Ca (77%) from the carbon surface, owing to the high solubility of the Ca(NO_3_)_2_ formed during the acid wash.

## Figures and Tables

**Table 1:** D_80_ (mm), iodine number (mg I_2_/g Ac), specific surface area (m^2^/g), and ball-pan hardens (%) of virgin carbon and spent activated carbon (SAC).

	Virgin Carbon	SAC
d_80_ (mm)	3.1	2.5
Iodine number (mg I_2_/g AC)	1036	734
Specific surface area (m^2^/g)	1012	869
Ball-pan hardness (%)	98.3	98.8

**Table 2:** Specific surface area (m^2^/g) of virgin carbon, SAC, and reactivated carbon.

Carbon	Specific Surface Area (m^2^/g)
Virgin carbon	1012
SAC	869
Reactivated carbon ^1^	1079

^1^ Reactivation conditions. Acid-wash: HNO_3_ 20% *v*/*v* at 50 °C. Thermal reactivation: 850 °C and 1 h.

**Table 3:** Iodine number (mg I_2_/g AC) of two sequences of reactivation (chemical-thermal and thermal chemical) for carbons subjected to an acid wash with HCl (10% *v*/*v* 50 °C), HNO_3_ (20% *v*/*v* 50 °C), H_2_SO_4_ (30% *v*/*v* 50 °C), and thermal reactivation during 1 h at 850 °C.

Carbon	Iodine Number (mg I_2_/g AC)	
	Chemical-Thermal	Thermal-Chemical
Acid wash: HCl 10% *v*/*v* 50 °C	1085	1035
HNO_3_ 20% *v*/*v* 50 °C	1199	1045
H_2_SO_4_ 30% *v*/*v* 50 °C	1128	1055

**Table 4:** Ball-pan hardness (%) and normal abrasion in pulp resisitance (%) of virgin carbon, SAC, chemically reactivated carbon, thermally reactivated carbon, and chemical-thermally reactivated carbon.

Carbon	Ball-Pan Hardness (%)	Normal Abrasion in Pulp Resistance (%)
Virgin carbon	98.3	92.5
SAC	98.8	97.1
Chemical reactivation ^1^	98.4	96.4
Thermal reactivation ^2^	97.3	90.1
Reactivated carbon ^3^	98.1	96.3

^1^ Acid wash: HNO_3_ 20% *v*/*v* 50 °C. ^2^ Thermal reactivation conditions: 850 °C and 1 h. ^3^ Reactivation conditions: acid-wash HNO_3_ 20% *v*/*v* at 50 °C and thermal reactivation at 850 °C and 1 h.

**Table 5:** Properties of virgin carbon, SAC, and reactivated carbon (moisture content (%), volatile content (%), ash content (%), fixed carbon (%), iodine number (mg I_2_/g AC), methylene blue index (mg/100 g AC), and sugar discoloration index (basic units of reference, UBR)).

Carbon	Virgin Carbon	SAC	Reactivated Carbon ^1^
Moisture content (%)	0.20	0.81	0.54
Volatile content (%)	5.27	8.35	6.10
Ash content (%)	3.09	12.08	3.35
Fixed carbon (%)	91.94	80.33	89.30
Iodine number (mg I_2_/g AC)	1036	734	1199
Methylene blue index (mg/100g AC)	27.91	27.84	27.90
Sugar discoloration index (UBR)	34.94	34.05	33.18

^1^ Reactivation conditions: acid-wash HNO_3_ 20% *v*/*v* at 50 °C and thermal reactivation at 850 °C and 1 h.

## Appendix A

### Elemental Analysis of Virgin Carbon and SAC

**Table A1:** Elemental analysis of virgin carbon and SAC.

Activated Carbon	C	H	N	O
	(%)			
Virgin	81.8	3.1	0.3	5.8
SAC	82.0	3.7	0.4	6.1

## Appendix C

### Characterization of the Auriferous Ore Used in the Normal Abrasion in the Pulp Resistance Test

The mineralogical characterization of the auriferous ore is presented in Table A2. This ore from the zone of Pacto in northern Ecuador has 21.45 g/ton of gold and 98.39 g/ton of silver.

**Table A2:** Mineralogical characterization of auriferous ore.

Mineral	Content (%)
Quartz	86.0
Pyrite	-
Galena	-
Clinochlore	7.2
Butlerite	2.1
Biotite	1.0
Chalcopyrite	-
Albite	3.7

## Appendix E

### FTIR Analysis of Virgin Carbon and SAC

**Table A3:** Interpretation of the FTIR spectrum of virgin carbon and SAC.

Functional Groups	Wavelength (cm^-1^)	
	Virgin Carbon	SAC
OH, water phenolic-hydroxylic group	3428	3400
Quinone, carboxylate	1571	1580
Aromatic ring	1600	1600
Carbonate	-	1383
Phenol	1100	1100
